# Chemical Vapor Deposition of 4 Inch Wafer‐Scale Monolayer MoSe_2_

**DOI:** 10.1002/smsc.202200062

**Published:** 2022-09-20

**Authors:** Jiawei Li, Shuopei Wang, Lu Li, Zheng Wei, Qinqin Wang, Huacong Sun, Jinpeng Tian, Yutuo Guo, Jieying Liu, Hua Yu, Na Li, Gen Long, Xuedong Bai, Wei Yang, Rong Yang, Dongxia Shi, Guangyu Zhang

**Affiliations:** ^1^ Songshan Lake Materials Laboratory Dongguan Guangdong 523808 China; ^2^ Beijing National Laboratory for Condensed Matter Physics Institute of Physics Chinese Academy of Sciences Beijing 100190 China; ^3^ School of Physical Sciences University of Chinese Academy of Sciences Beijing 100190 China

**Keywords:** chemical vapor deposition, field-effect transistors, monolayer MoSe_2_, transition metal dichalcogenides, wafer-scale monolayers

## Abstract

2D semiconducting transition metal dichalcogenides (TMDs) are considered promising building blocks for emergent electronic and optoelectronic devices. As one of the representatives of 2D semiconductors, monolayer MoSe_2_ has excellent electrical and optical properties and has attracted a lot of research interest recently. To realize various device applications, large‐scale synthesis of monolayer MoSe_2_ with high crystal quality is critical, yet remains challenging. Herein, the growth of monolayer MoSe_2_ at a 4 inch wafer‐scale by chemical vapor deposition is demonstrated. Based on a multisource design and vertical placement of substrates, wafer‐scale continuity and uniformity of layer thickness, e.g., the monolayer, are achieved. This growth technique is also applicable to the wafer‐scale growth of other 2D semiconductors such as WS_2_ and MoS_2_.

## Graphical abstract

A 4 inch wafer‐scale monolayer MoSe_2_ is successfully synthesized by chemical vapor deposition. Based on a multisource design and vertical placement of substrates, the monolayer films achieve wafer‐scale continuity and uniformity, which suggest a great potential for electronic devices. This growth technique is also applicable to the wafer‐scale growth of other 2D semiconductors families such as WS_2_ and MoS_2_.

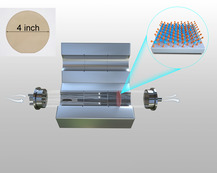

## 1 Introduction

2D transition metal dichalcogenides (TMDs) have excellent electrical and optical properties,^[^ ^1^, ^2^, ^3^ ^]^ offering many possibilities to revolutionize existing semiconductor technologies based on bulk materials or their thin films. In the 2D TMD family, monolayer MoSe_2_ has gained considerable attention for various optoelectronic, electrochemical, and photocatalytic applications. For example, it has a direct bandgap of ≈1.55 eV, which is close to the optimal value for solar cells.^[^ ^4^ ^]^ The unsaturated Se atoms at its edges and defect sites could induce electrocatalytic activity toward hydrogen evolution reaction and similar electrocatalytic reactions such as in lithium‐oxygen batteries.^[^ ^5^ ^]^ In addition, monolayer MoSe_2_ film could be applicable to the spintronic devices due to its large spin splitting energy of ≈180 meV at the top of the valence band.^[^ ^6^, ^7^ ^]^ Compared with the widely studied MoS_2_, MoSe_2_ has higher optical quality^[^ ^8^, ^9^ ^]^ and ambipolar characteristics in field‐effect transistors (FETs)^[^ ^10^, ^11^ ^]^ which may lead to better‐functionalized devices in both electronics and optoelectronics.

To realize these applications, the preparation of large‐area and uniform MoSe_2_ films becomes particularly important. Top‐down methods like mechanical exfoliation and chemical exfoliation^[^ ^12^, ^13^ ^]^ are not able to achieve large‐scale uniform monolayer films. With the development of bottom‐up methods, various techniques have been developed to synthesize monolayer MoSe_2_ so far, including chemical vapor deposition (CVD),^[^ ^10^, ^14^, ^15^, ^16^, ^17^, ^18^, ^19^ ^]^ metal–organic chemical vapor deposition (MOCVD),^[^ ^20^ ^]^ molecular beam epitaxy (MBE),^[^ ^11^ ^]^ atomic‐layer deposition (ALD),^[^ ^21^ ^]^ and selenization of metal or metal compounds.^[^ ^22^ ^]^ In general, selenium has a lower reaction activity than sulfur, the growth of monolayer MoSe_2_ is more difficult than that of monolayer MoS_2_,^[^ ^23^, ^24^, ^25^ ^]^ and as‐grown monolayer MoSe_2_ at a large scale is usually not uniform.^[^ ^10^, ^15^, ^26^, ^27^, ^28^, ^29^ ^]^

In this work, we report the growth of monolayer MoSe_2_ continuous films at a 4 inch wafer scale, the largest ever achieved. The wafer‐scale uniformity is facilitated by the design of multisources and the vertical loading of substrates in the CVD system. Structural, spectroscopic, and electrical characterizations on the monolayer MoSe_2_ films reveal the high crystalline quality and wafer‐scale uniformity. We also demonstrated the universality of the designed CVD system in other TMD families, including MoS_2_ and WS_2_, shedding light on its potential applications in electronics and optoelectronics.

## 2 Results and Discussion

**Figure 1:**
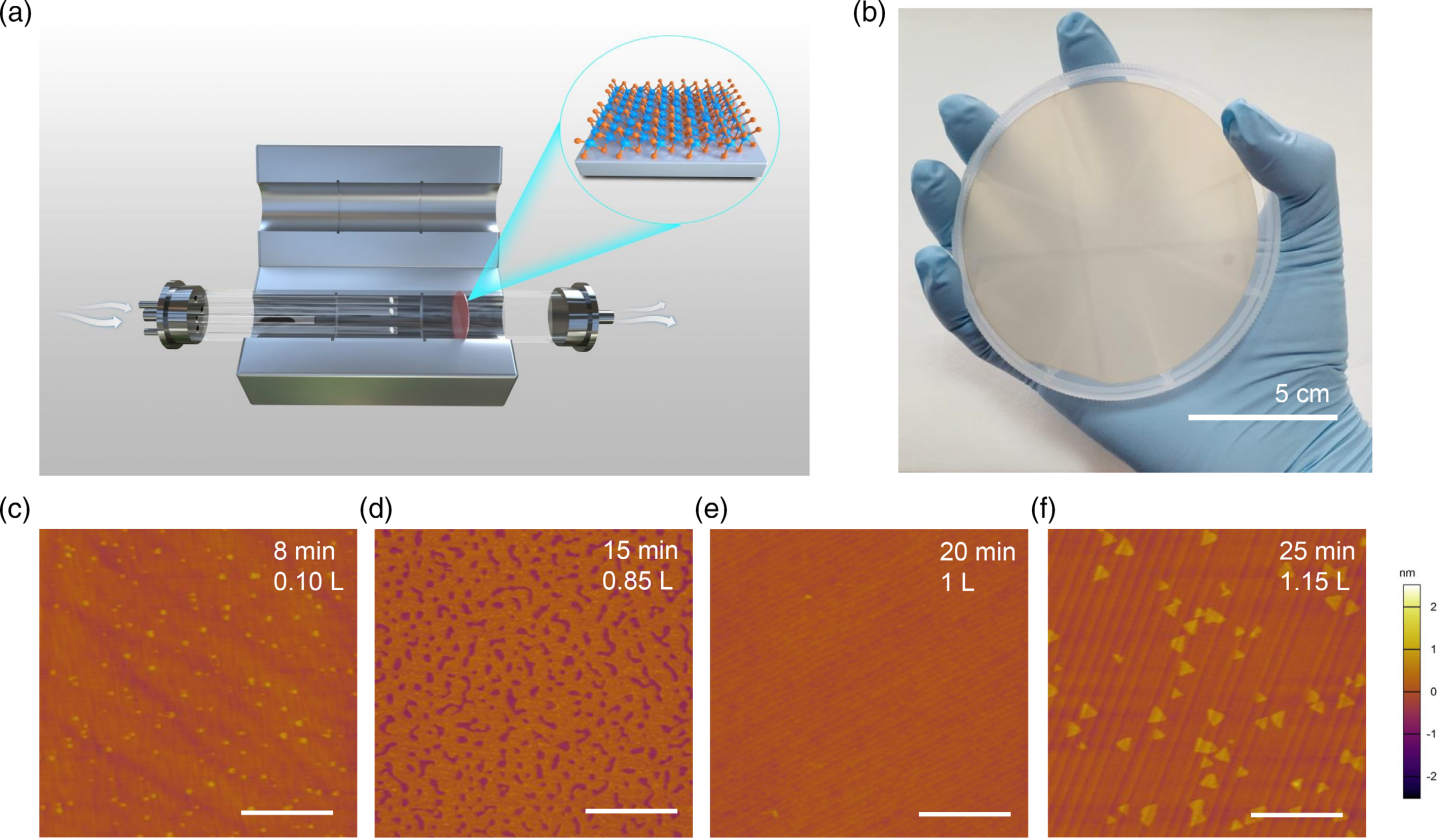
Chemical vapor deposition (CVD) growth of 4 inch wafer‐scale MoSe_2_. a) Schematic diagram of CVD system. b) Photo of 4 inch wafer monolayer MoSe_2_ on sapphire substrate. c–f) Atomic force microscopy (AFM) images of MoSe_2_ with different coverage in different growth times. Scale bar: 1 μm.

The 4 inch wafer‐scale monolayer MoSe_2_ films are grown in a CVD system equipped with a three‐temperature‐zone tube furnace (**Figure** **1**a). Se and MoO_3_ powders are loaded in the first and second temperature zones, respectively; the sapphire substrate is loaded in the third temperature zone. As seen in Figure 1a, three small quartz tubes in the growth chamber serve as containers for the MoO_3_ sources, and each tube is delivered independently with a mixture of argon and hydrogen (7% H_2_) as carrier gases. This multisource design provides the homogeneous cross‐sectional source supply of MoO_3_ and is the key to uniform growth at the wafer scale. Usually, the gradient of precursor concentration and temperature along the horizontal direction led to nonuniform growth of the TMDs.^[^ ^30^, ^31^ ^]^ This position‐dependent growth is more pronounced if the substrate scales to 4 inch (Figure S1, Supporting Information). The as‐grown wafer shows thickness uniformity which is visible even to the naked eye, similar to previous reports.^[^ ^28^ ^]^ To avoid this phenomenon in this work, the 4 inch sapphire wafer is vertically placed in the growth chamber. We performed a computational fluid dynamics (CFD) simulation to study the distribution of gas flow velocity in the CVD system (Figure S1–S3, Supporting Information). From CFD simulations, we can clearly identify that the vertically placed substrate has a much more uniform gas flow distribution of precursor on its surface than the horizontally placed substrate.

Benefiting from the multisource design and vertical substrate loading, we thus can reliably grow uniform MoSe_2_ monolayers on C‐plane sapphire (0001) over the entire 4 inch wafer surface (Figure 1b). A typical growth time for the completion of a 4 inch monolayer MoSe_2_ lasts 20 min, as illustrated in Figure S4 (Supporting Information). The 4 inch wafer size achieved here is the largest ever reported on the growth of monolayer MoSe_2_ on either SiO_2_ or sapphire (Table S1, Supporting Information). It is worth noting that most of the 2D materials that have been successfully grown at the wafer scale share a similar route: 1) nucleation of 2D seeds; 2) lateral growth of these seeds into large domains; and 3) coalescence of 2D domains into a continuous film.^[^ ^32^ ^]^ In this work, the monolayer MoSe_2_ nucleates at multiple sites on the sapphire substrate. Due to the multisource design, MoSe_2_ nucleates uniformly on the entire wafer, and the nucleation density is relatively high, so the wafer‐scale monolayer continues film can be grown in a short time. Figure 1c–f shows the atomic force microscopy (AFM) images of as‐grown MoSe_2_ with different growth times. It can be seen that MoSe_2_ grains nucleate on the sapphire substrate, grow gradually into larger sizes, and eventually merge into a complete monolayer film with an average grain size of a few hundreds of nm. Figure S5 (Supporting Information) shows the zoomed‐in AFM images of the monolayer MoSe_2_ film. The film is continuous and clean. Hydrogen is introduced during the growth, leading to the etching of the sapphire substrate and producing oxygen‐vacancy defects, which increases the nucleation density.^[^ ^33^, ^34^ ^]^ To increase the grain size, we controlled the inflow time and flow rate of hydrogen, and we also optimized the growth parameters such as substrate temperature and the distance between source and substrate (Figure S6, Supporting Information) for the continuous monolayer film growth. When further extending the growth time, the second and third layers of MoSe_2_ flake with triangular shapes would appear (Figure S7, Supporting Information). Note that, during the growth of MoSe_2_ domains, the coming adatoms from sources would strongly bond to the domain edges by forming chemical bonds and weakly bonded to the sapphire substrate and the domain surfaces by forming van der Waals bonds. Thus, the 2D growth mode is energetically preferable to the 3D growth mode for monolayer 2D film growth on the sapphire substrate.^[^ ^35^, ^36^ ^]^ This energy benefit facilitates wafer‐scale continuity and uniformity.

**Figure 2:**
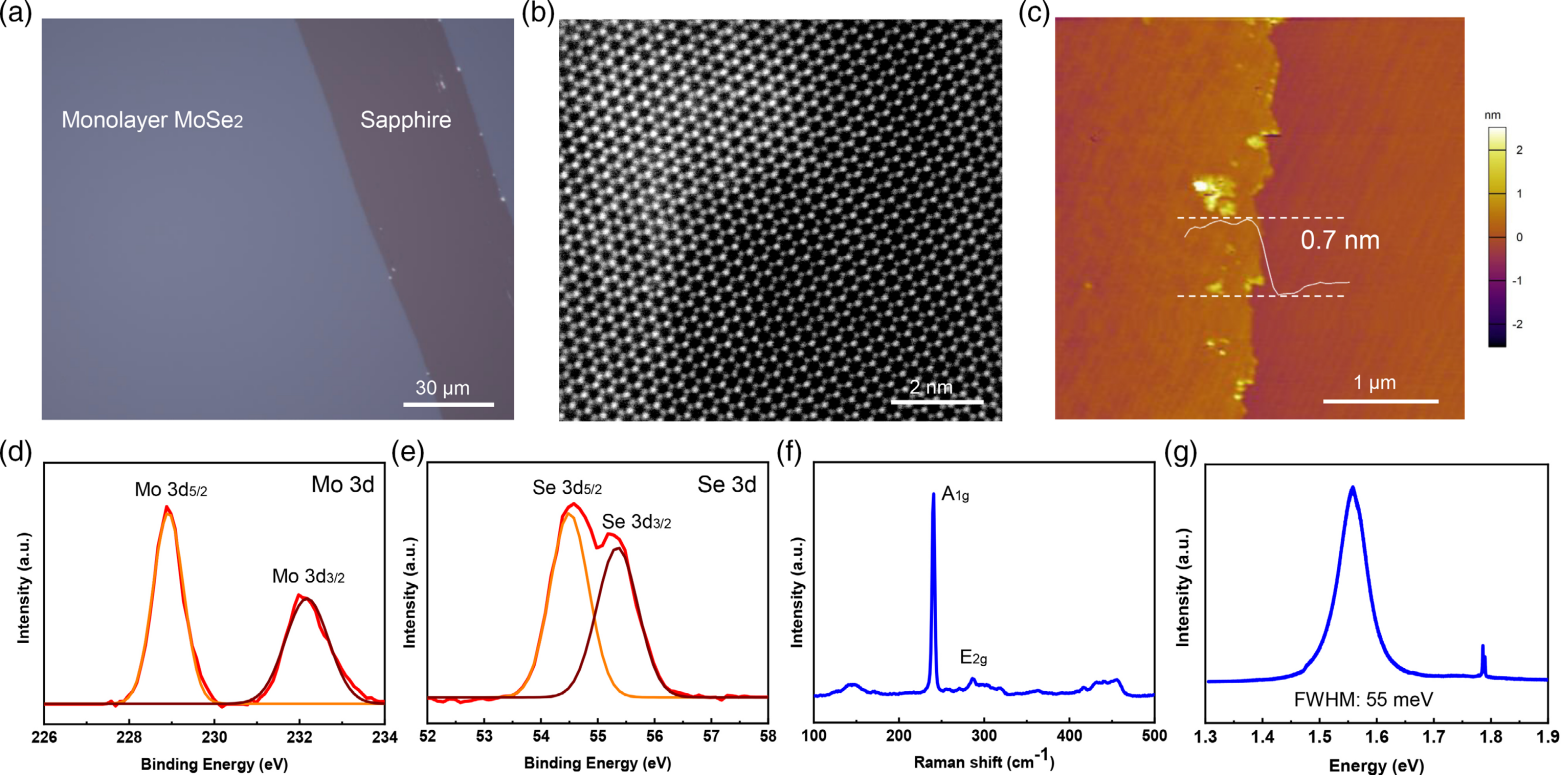
Characterization of structure and element composition of MoSe_2_. a) OM image of monolayer MoSe_2_ film on a sapphire substrate with an intentional scratch on the right side. b) STEM image of monolayer MoSe_2_. c) AFM image of monolayer MoSe_2_ and the height profile in the AFM image. d,e) X‐ray photoelectron spectroscopy (XPS) spectra of Mo 3d and Se 3d of monolayer MoSe_2_. f,g) Raman spectra and photoluminescence (PL) spectra of monolayer MoSe_2_.

**Figure** **2**a shows an optical microscopy (OM) image of an as‐grown monolayer MoSe_2_ film on sapphire. The lattice structure within the MoSe_2_ domains is characterized by the scanning transmission electron microscopy (STEM). An atomic‐resolution high‐angle annular dark‐field (HAADF) image is shown in Figure 2b. We can see that the monolayer MoSe_2_ is of 1H‐phase and nearly perfect hexagonal lattice with negligible Se vacancies observed. The selected‐area electron diffraction (SAED) patterns of the monolayer MoSe_2_ film show multiple patterns (Figure S8, Supporting Information), suggesting the polycrystalline nature of the film. The thickness of the MoSe_2_ film is ≈0.7 nm, indicating that the film is monolayer (Figure 2c). X‐ray photoelectron spectroscopy (XPS) was performed to examine the elemental composition of the MoSe_2_ film. The peaks of Mo 3d_3/2_ and 3d_5/2_ core levels are located at 232.1 and 228.9 eV, respectively (Figure 2d). The signals of MoO_3_ were completely absent, indicating that the Mo^6+^ in MoO_3_ was reduced to Mo^4+^. The peaks of Se 3d_3/2_ and 3d_5/2_ core levels are located at 54.6 and 55.3 eV, respectively (Figure 2e). These data are consistent with the results previously reported,^[^ ^19^, ^26^ ^]^ and the integrated peak areas indicate that the stoichiometry of the Mo/Se ratio is close to 1:2, demonstrating that the obtained material is MoSe_2_. Figure 2f shows the Raman spectra of the as‐grown monolayer MoSe_2_ film. Two characteristic peaks of MoSe_2_ are located at 240 cm^−1^ and 287 cm^−1^, which are attributed to the out‐of‐plane vibrations (A_1g_ mode) and in‐plane vibrations (E_2g_ mode), respectively.^[^ ^19^, ^37^ ^]^ Figure 2g shows the photoluminescence (PL) spectra of the monolayer MoSe_2_ film with a strong PL peak at 790 nm, indicating the direct bandgap of 1.57 eV of the monolayer MoSe_2_. The full width at half maxima (FWHM) is 55 meV, which is comparable to that of mechanically exfoliated monolayer MoSe_2_ (Figure S9, Supporting Information).

**Figure 3:**
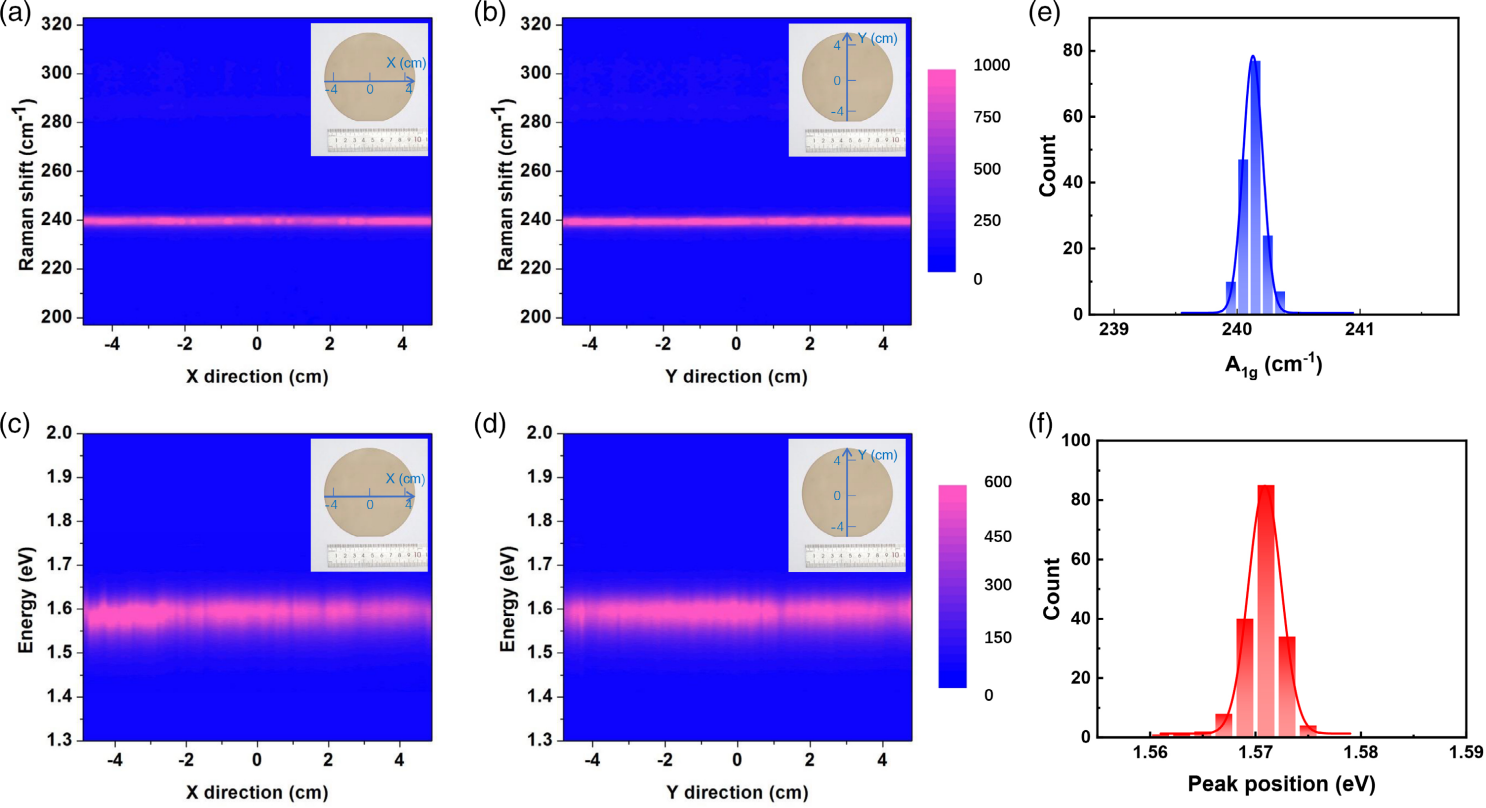
Uniformity of wafer‐scale monolayer MoSe_2_. a–d) Raman and PL line scan mapping along the diameter. e) Statistical distributions of the A_1g_ peak position extracted from the Raman spectra in (a,b). f) Statistical distributions of PL peak position extracted from the PL spectra in (c,d).

To characterize the uniformity of the as‐grown 4 inch monolayer MoSe_2_ films, we thus performed Raman and PL line scans along both the horizontal axis (*X*) and vertical axis (*Y*) across the wafer. The spectra obtained from line scans were nearly identical to each other for both Raman and PL. As the intensity of the A_1g_ peak is much stronger than the E_2g_ peak, the E_2g_ peak can hardly be seen. The statistics of the Raman and PL spectra obtained from line scans shows that the average A_1g_ peak is 240.12 cm^−1^ with a standard deviation of 0.086 cm^−1^ (**Figure** **3**e) and the average PL position is 1.57 eV with a standard deviation of 2 meV (Figure 3f). The spatial Raman and PL mapping (Figure S10, Supporting Information) also show the homogeneity of grown monolayer MoSe_2_ continuous film. Besides, OM images and AFM images of our monolayer MoSe_2_ wafers in different areas are also shown in Figures S11 and S12 (Supporting Information), respectively, confirming the wafer‐scale uniformity of the monolayer MoSe_2_ film.

Other than MoSe_2_, our method was also applicable to other TMD monolayers, such as MoS_2_ and WS_2_ at the wafer scale (Figure S13, Supporting Information). For the batch production capability, the maximum sample size is only limited by the diameter of the tube furnace; a larger sample size is reachable by increasing the size of the furnace. Based on this large‐scale monolayer growth technique, we also investigated the wafer‐scale growth of lateral heterostructures which have recently attracted great research interest.^[^ ^38^, ^39^, ^40^, ^41^, ^42^ ^]^ To achieve this goal, we first grow randomly distributed monolayer triangular domains of WS_2_ or MoS_2_ on the sapphire substrate by CVD, and then grow MoSe_2_ in a subsequent growth step. Compared with the already‐formed WS_2_ or MoS_2_, sapphire has much higher surface energy; thus, the MoSe_2_ monolayers preferentially grow on the sapphire substrate rather than on already‐formed WS_2_ or MoS_2_ domains. After sufficient growth of MoSe_2_, a fully covered and continuous film of lateral heterostructures can be achieved, as shown in Figure S14 (Supporting Information). From the OM and the AFM images (Figure S14a–d, Supporting Information), the lateral heterostructure is very smooth and no obvious impurity adsorption or thick layers grown at the boundary are observed. From the high‐resolution HAADF image and the line intensity profiles (Figure S14f–g, Supporting Information), the lateral heterostructure has a clear boundary, and there is no binary alloy, structural damage, or phase transition along the boundary.

**Figure 4:**
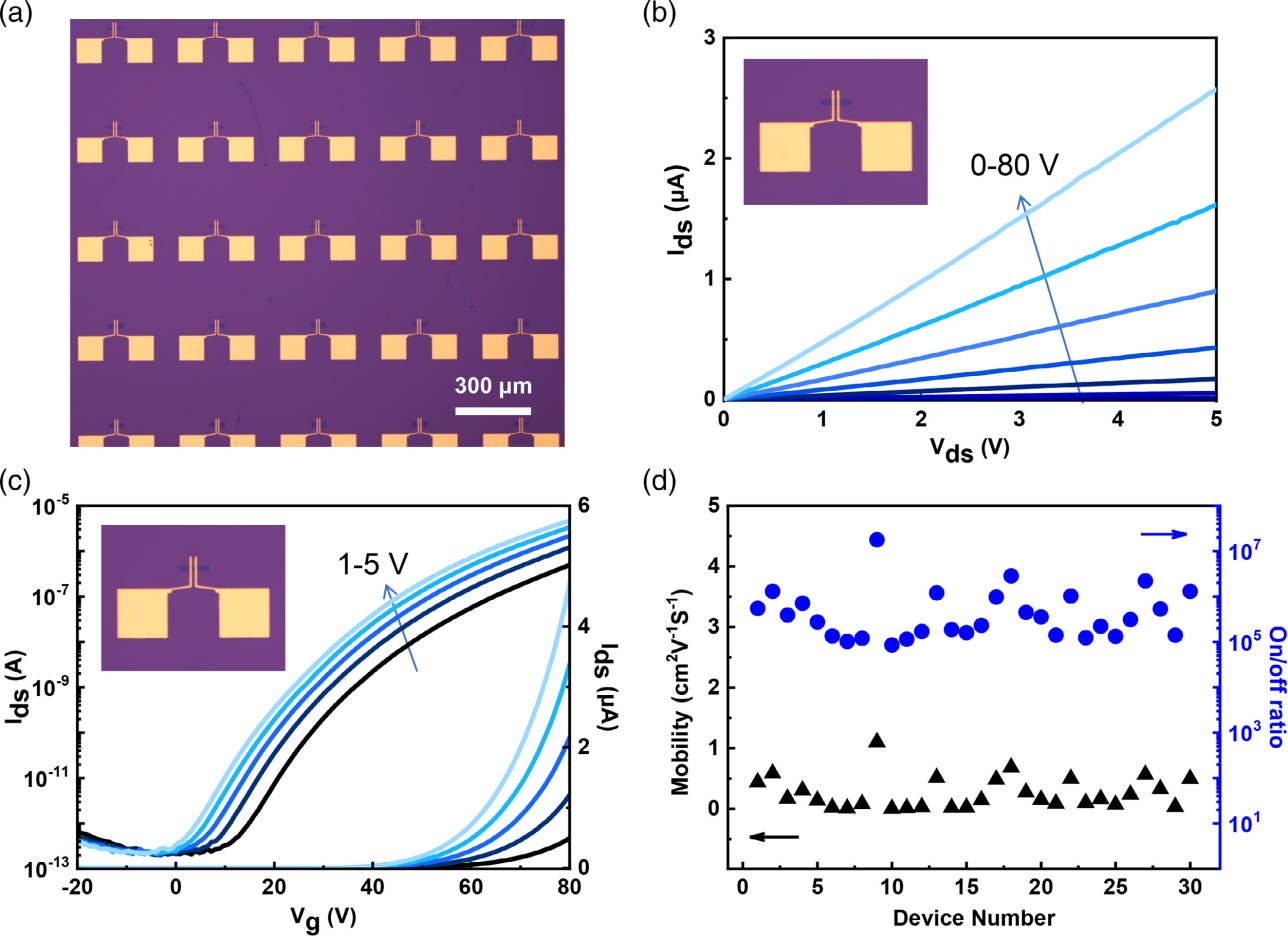
Electrical properties of monolayer MoSe_2_ film. a) OM image of MoSe_2_ FET array. b) Typical output characteristics of MoSe_2_ FET at *V*_g_ = 0–80 V. c) Typical transfer characteristics of MoSe_2_ FET at *V*_ds_ = 1–5 V. d) The mobility and on/off ratio of 30 MoSe_2_ FETs.

Such 2D semiconductor wafers are promising for next‐generation memories and logic circuits.^[^ ^43^, ^44^ ^]^ Thus, we characterized the electrical properties of the monolayer MoSe_2_ films. As‐grown monolayer MoSe_2_ films are transferred to the 300 nm thick SiO_2_/Si substrates for back‐gate FET device fabrications. **Figure** **4**a shows the OM image of the monolayer MoSe_2_ FETs array with a channel length of 5 μm. Figure 4b,c shows the output and transfer curves of a typical MoSe_2_ FET with the channel width/length of 10/5 μm, suggesting an n‐type behavior. Figure 4d shows the statistics on the field‐effect mobility and on/off ratios of 30 FET devices. These devices exhibit reasonably good uniformity with an on/off ratio of ≈10^6^ and field‐effect mobility of ≈1 cm^2^ V^−1 ^s^−1^. The device performances achieved from MoSe_2_ are much lower than that from MoS_2_ and further improvements are awaiting, e.g., optimizing the device structure and engineering the interface.

## 3 Conclusion

We have achieved CVD growth of 4 inch wafer‐scale monolayer MoSe_2_ films. Guided by CFD simulations, we designed a multisource CVD system combined with the vertical loading of substrates. Due to the homogeneous cross‐sectional source supply, the monolayer MoSe_2_ films are uniform across the entire wafers. Our work provides a general strategy for the large‐scale growth of 2D TMDs and their lateral heterostructures, shedding light on the cost‐effective batch production of 2D semiconductors.

## 4. Experimental Section

1

1.1

##### CVD Growth of Monolayer MoSe_2_ Film on Sapphire

The 4 inch monolayer MoSe_2_ growth was performed in a low‐pressure chemical vapor deposition system. The CVD system was manufactured by Dongguan Join Technology Co., Ltd. Se (Alfa Aesar, 99.5%) was loaded in the center quartz tube and placed at zone1. MoO_3_ (Alfa Aesar, 99.998%) powders were loaded in three separate quartz tubes and placed at zone2. The 4 inch sapphire substrate was first annealed at 1,000 °C in Ar/O_2_ atmosphere for 4 h, and placed in zone3. The growth temperature for each zone was 280, 420, and 930 °C, respectively. The carrier gas was Ar/H_2_ (600/50 sccm). The pressure was 1–5 Torr during the growth. For a typical growth, the growth process lasted for 10–30 min.

##### CVD Growth of Monolayer MoS_2_, WS_2_ Film on Sapphire

The growth recipes were very similar to that for MoSe_2_ except for the replacement of MoO_3_ by WO_3_ source and Se by S source. The temperature settings were adjusted accordingly: 900 °C for WO_3_ and 120 °C for S in the growth process.

##### Structural and Spectroscopic Characterizations

AFM images were performed by Asylum Research Cypher S. STEM images and SAED patterns were performed by the JEM‐ARM300F (JEOL) operated at 80 kV. Raman and PL characterizations were performed by a Horiba Jobin Yvon LabRAM HR‐Evolution Raman system with a laser excitation wavelength of 532 nm. XPS characterizations were performed by Thermo Fisher Scientiﬁc ESCALAB 250X.

##### Device Fabrication and Measurements

The grown MoSe_2_ film was transferred to Si/SiO_2_ substrate by wet transfer method assisted with poly(methyl methacrylate). The back‐gate FET devices were fabricated using UV lithography (MA6, Karl Suss), reactive ion etching (Plasma Lab 80 Plus, Oxford Instruments), and electron beam evaporation of Ti/Au (3 nm/30 nm) source/drain electrodes. The device measurements were carried out in a probe station with semiconductor parameter analyzers (Agilent B1500).

## Conflict of Interest

The authors declare no conflict of interest.

## Author Contributions

G.Z. supervised this research. J.L. and S.W. performed the MoSe_2_ growth, AFM and Raman characterization, and device fabrication, Z.W. performed XPS characterization, H.S. performed TEM characterization, L.L., Q.W., J.T., Y.G., J.L., N.L., X.B., L.G., W.Y., R.Y., D.S., and G.Z. analyzed the data; J.L., S.W., and G.Z wrote, and all authors commented on the manuscript.

## Supporting information

Supplementary Material

## Data Availability

The data that support the findings of this study are available from the corresponding author upon reasonable request.
