# Supplementary material for: Chemical Vapor Deposition of 4 Inch Wafer‐Scale Monolayer MoSe2
Source: Small Sci. 2022 Sep 20;2(11):2200062. doi: 10.1002/smsc.202200062 (PMC11935850; doi:10.1002/smsc.202200062)
Supplement: Supplementary file 1 — Supplementary Material [file SMSC-2-2200062-s001.pdf]

Supporting Information

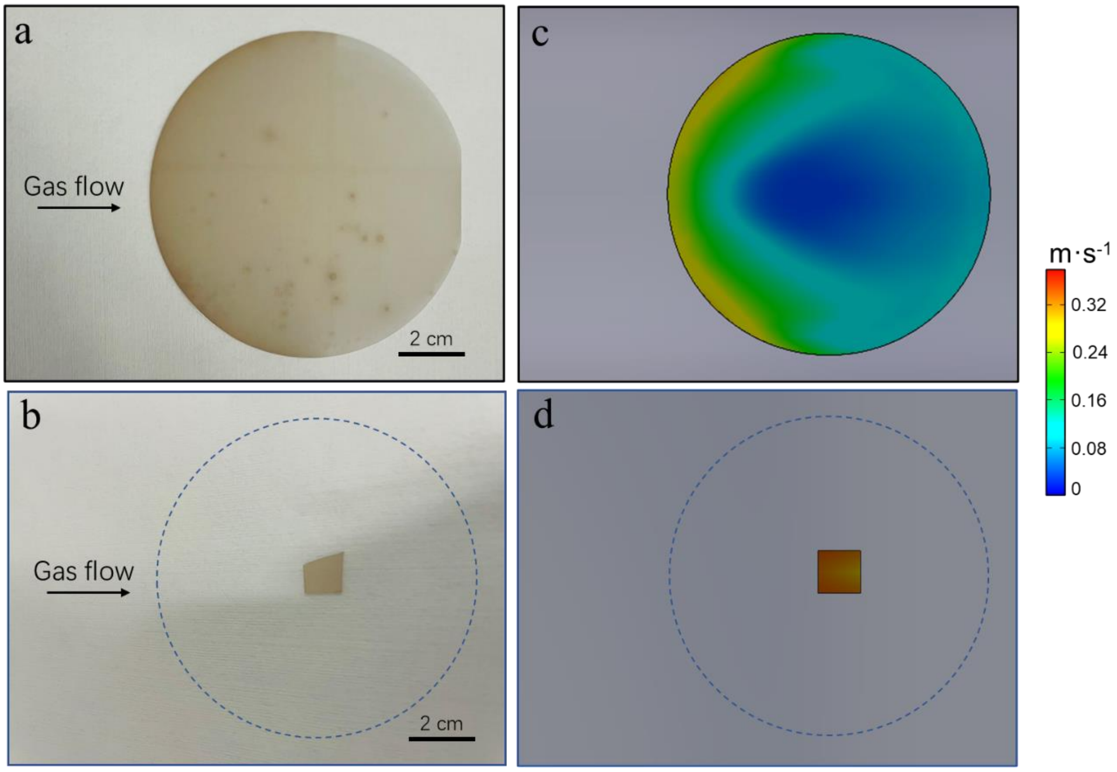

Figure S1. a) Photograph of 4-inch wafer grew in a horizontal CVD system, which shows non-uniform growth at wafer-scale. b) Photograph of a 1x1 cm^2 substrate, which was placed in the center. The latter shows more uniform coverage when substrate get smaller. c,d) Corresponding CFD simulation of the distribution of gas flow velocity in surface of sample.

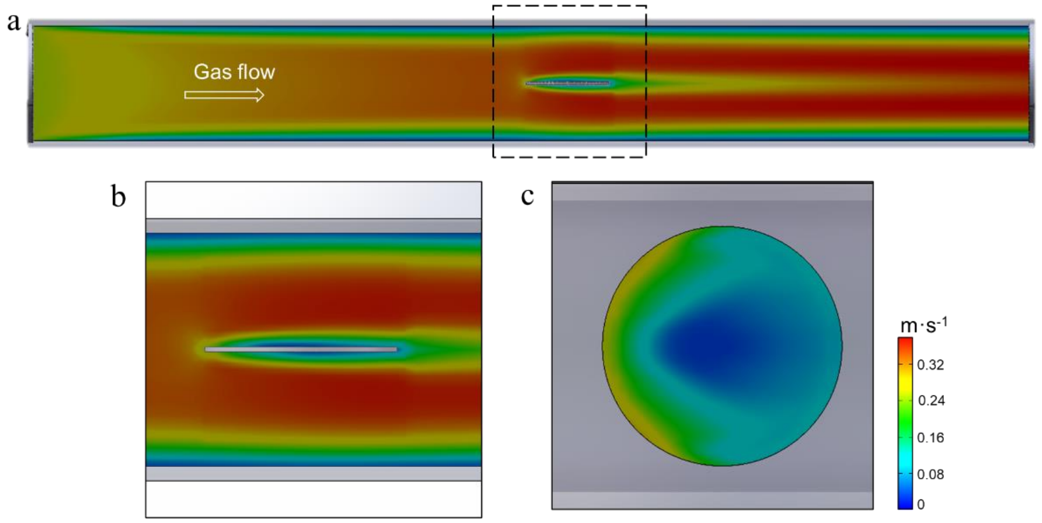

Figure S2. Setup of typical CVD system. a) CFD simulation of the distribution of gas flow velocity in typical CVD systems. b) Details corresponding to the rectangular zone marked in (a). c) CFD simulation of the distribution of gas flow velocity in surface of sample.

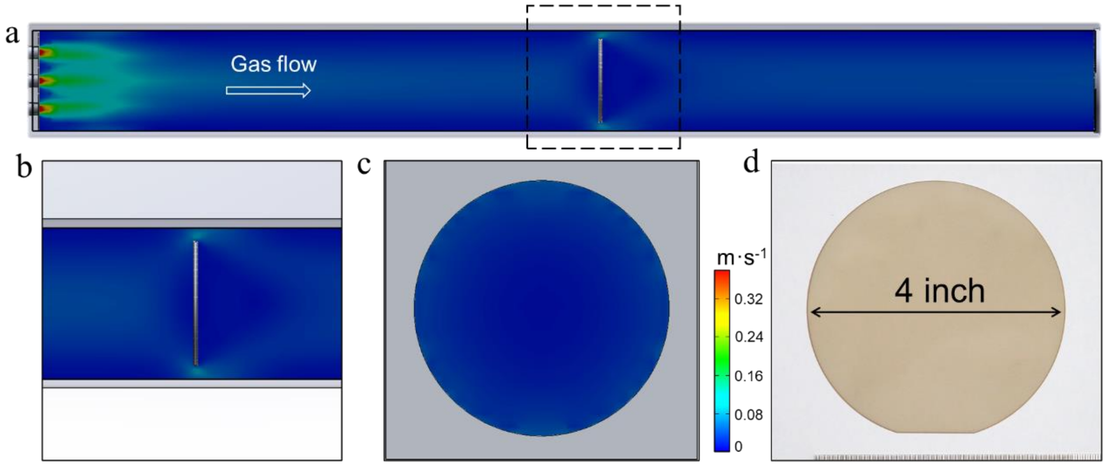

Figure S3. Setup of the designed CVD system. a) CFD simulation of the distribution of gas flow velocity in the designed systems. b) Details corresponding to the rectangular zone marked in (a). c) CFD simulation of the distribution of gas flow velocity in surface of sample. d) Photograph of as-grown 4-inch monolayer $MoSe_2$ on sapphire substrate.

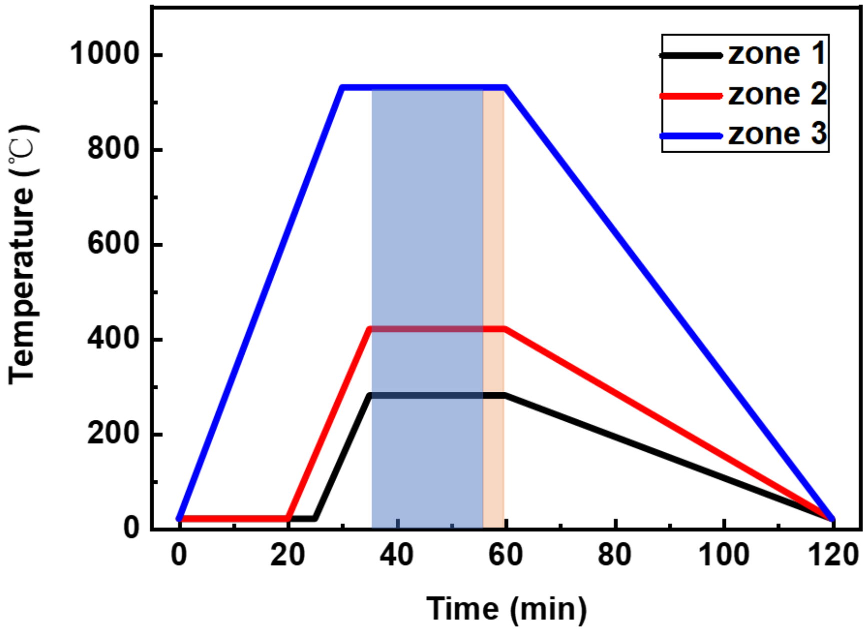

Figure S4. Temperature ramp used during the growth process. 20 minutes for growth (blue area) and 5 minutes for annealing (red area).

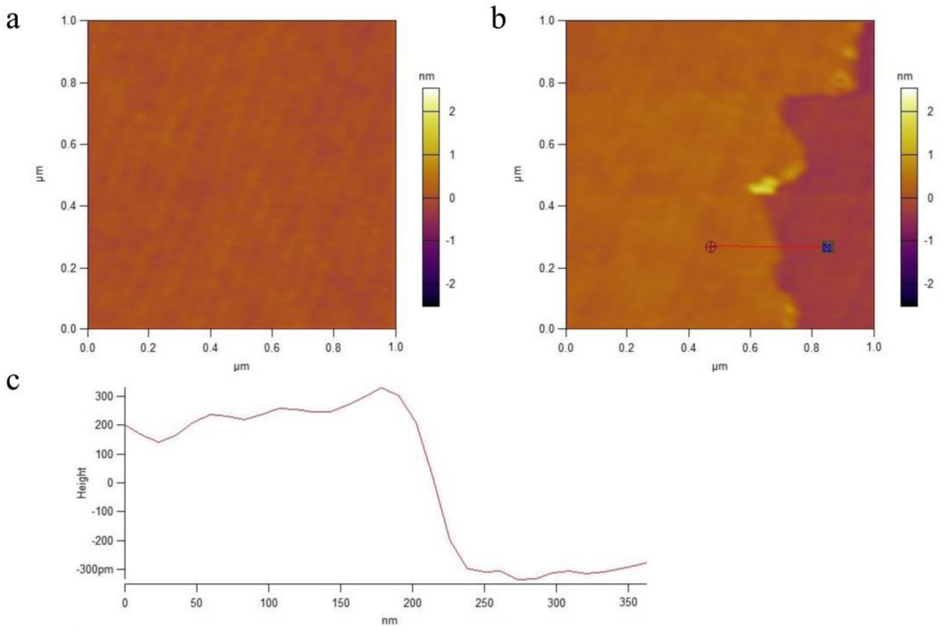

Figure S5. a) AFM image of monolayer $MoSe_2$ film. b) AFM image of monolayer $MoSe_2$ film with a scratch at right side. c) Height profiles of the $MoSe_2$ film along the red line in b).

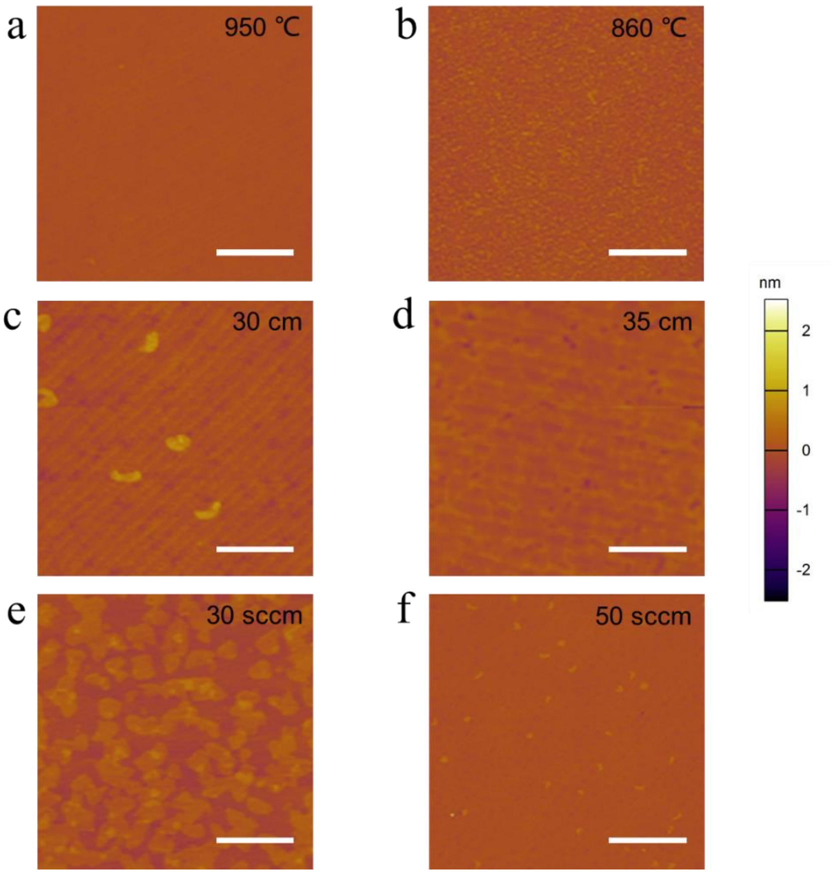

Figure S6. AFM images of $MoSe_2$ under different growth conditions. The temperatures of substrate were 950 ℃ a) and 860 ℃ b), respectively. The source to substrate distances were 30 cm c) and 35 cm d), respectively. The gas flow rate of H_2 were 30 sccm e) and 50 sccm f), respectively. Scale bar: 1 μm.

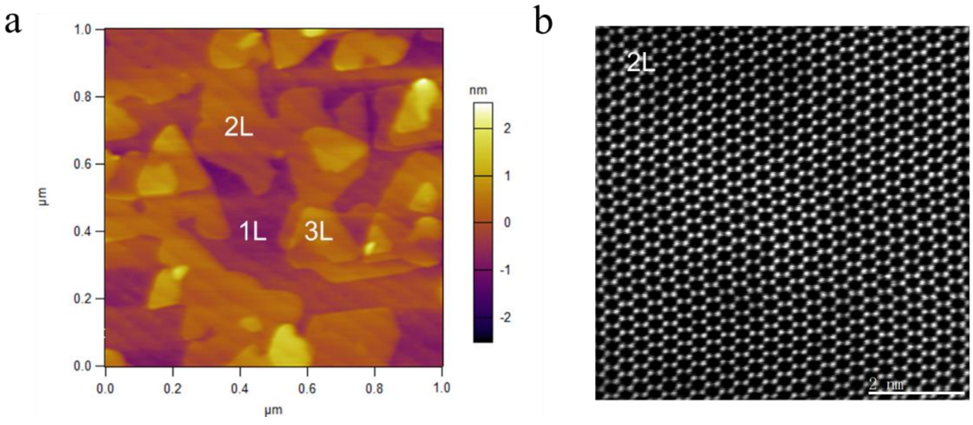

Figure S7. a) AFM image of $MoSe_2$ with 1-3 layers. b) STEM image of bilayer $MoSe_2$. Scale bar: 2 nm.

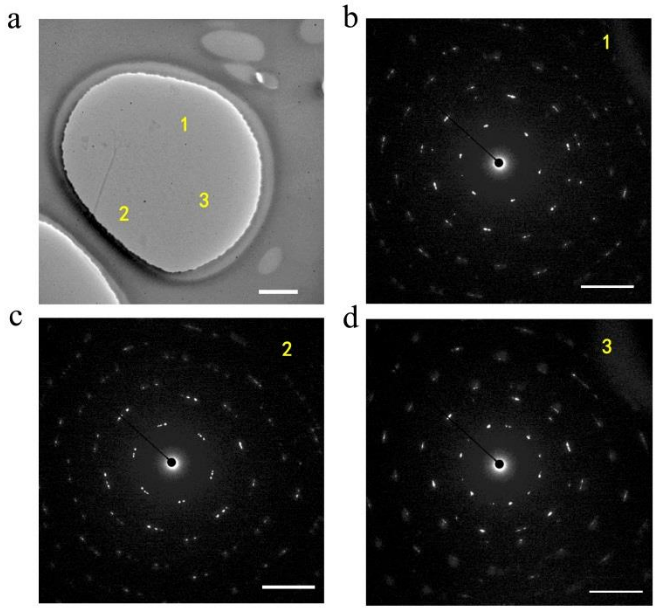

Figure S8. a) TEM image of a transferred $MoSe_2$ film on a TEM grid. Scale bar: 500 nm. b-d) SAED patterns of position1 (b), 2 (c) and 3 (d) on the monolayer $MoSe_2$ film, respectively. Scale bar: 5 nm^{-1}.

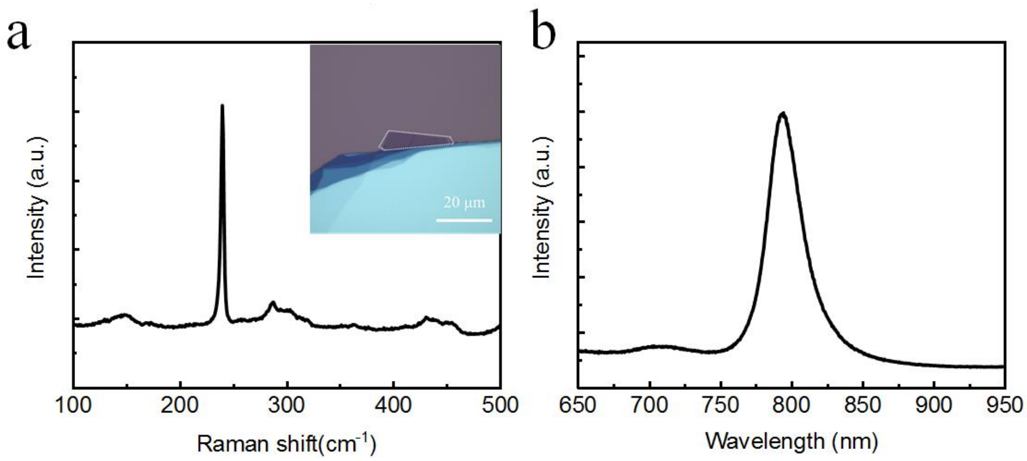

Figure S9. a,b) Raman and PL spectra of mechanically exfoliated monolayer $MoSe_2$ on SiO_2.The monolayer area was highlighted by the dash line.

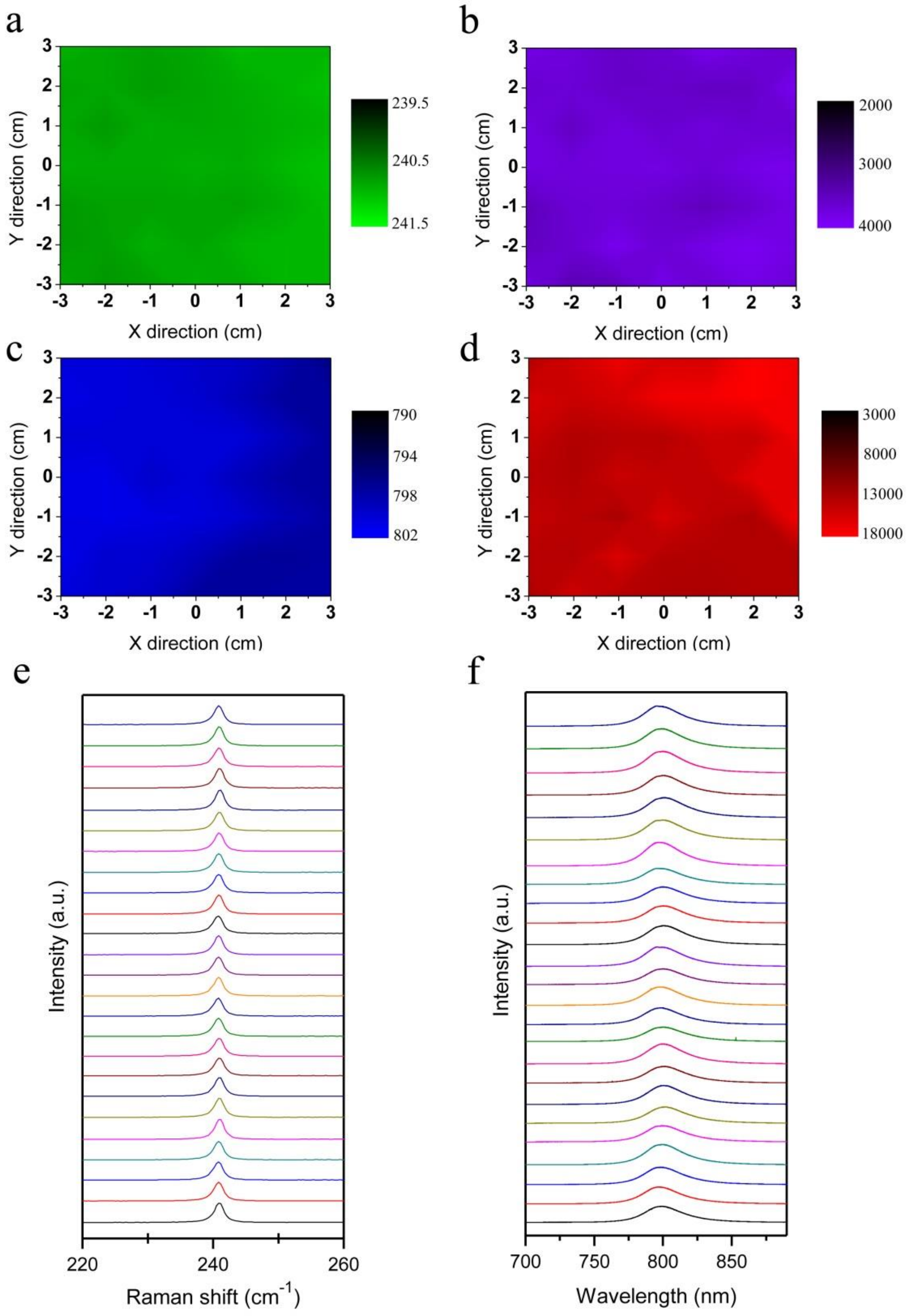

Figure S10. a) The Raman mapping of A1g peak position. b) The Raman mapping of A1g peak intensity. c) The PL mapping of PL peak position. d) The PL mapping of PL peak intensity. e, f) Raman and PL spectra of the $MoSe_2$ film obtained at 25 different regions.

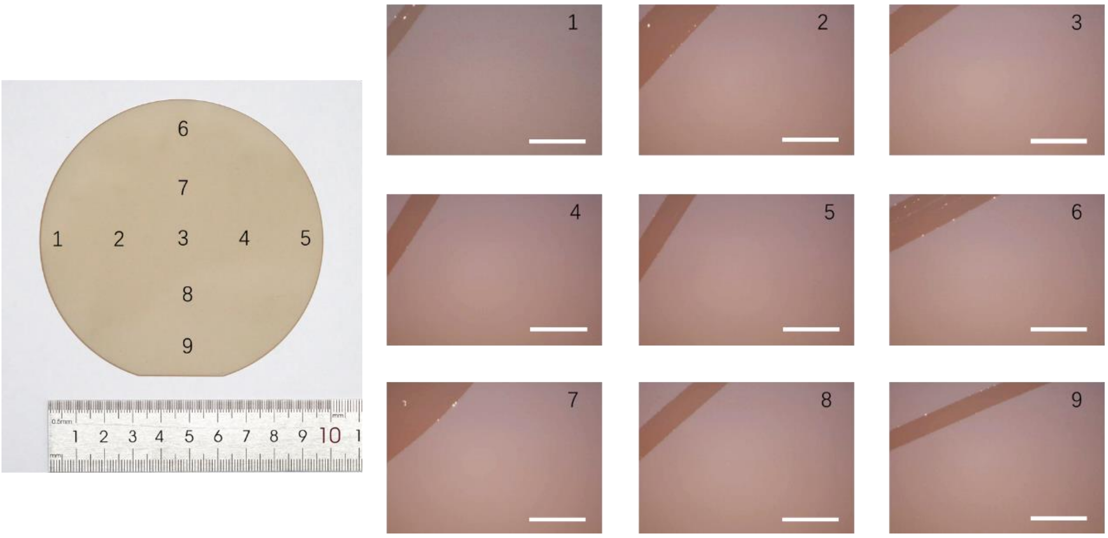

Figure S11. OM images of different positions of a monolayer $MoSe_2$ film grown on a 4 in. sapphire wafer. Scale bar: 30 μm.

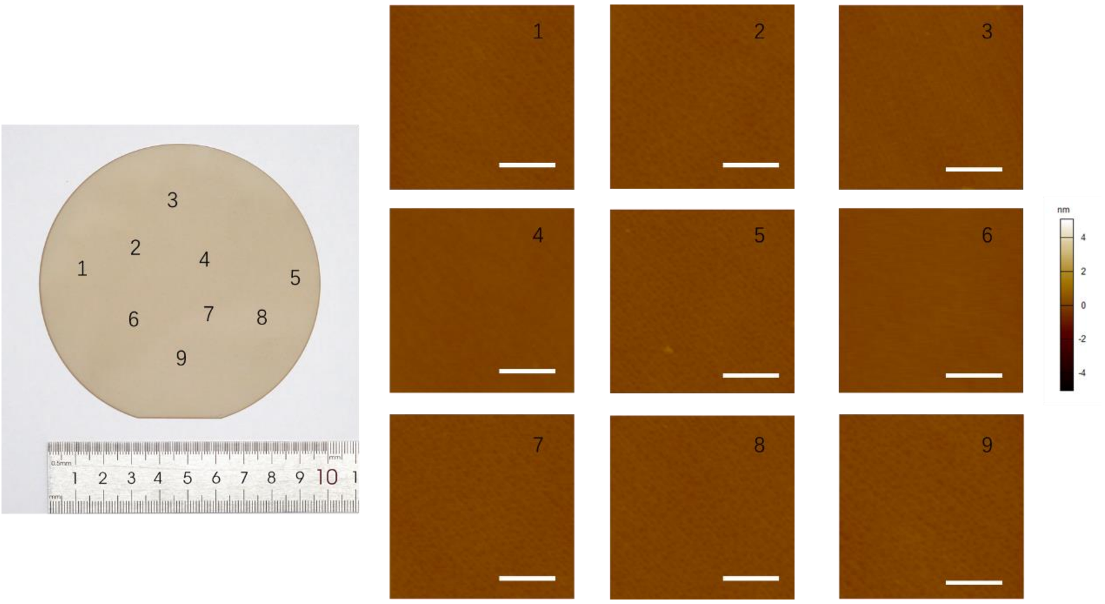

Figure S12. AFM images of different positions of a monolayer $MoSe_2$ film grown on a 4 in. sapphire wafer. Scale bar: 1 µm.

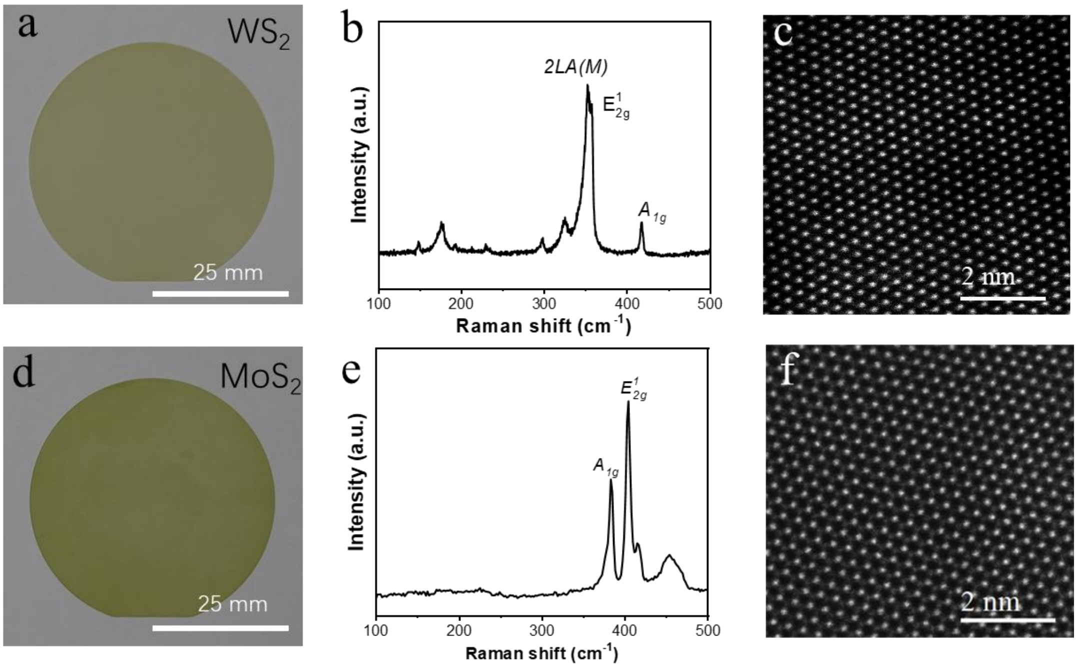

Figure S13. Characterization of the obtained 2-inch WS_2, MoS_2 wafer. a,d) Optical images, b,e) Raman spectra, and c,f) STEM-HADDF images of WS_2, MoS_2**.**

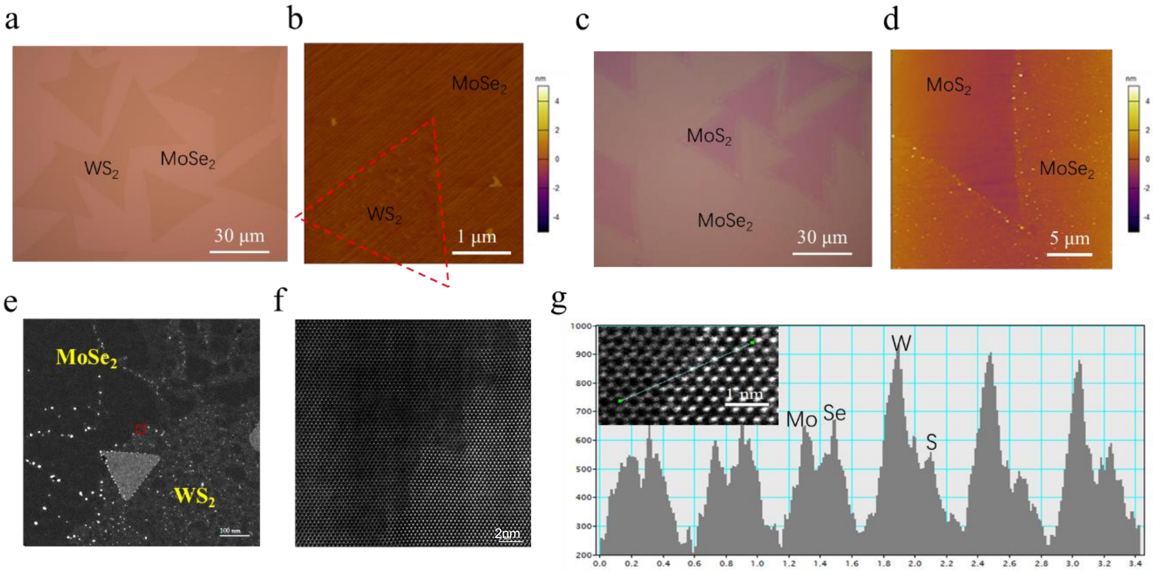

Figure S14. WS_2/$MoSe_2$ and MoS_2/$MoSe_2$ lateral heterostructure. a) OM image of WS_2/$MoSe_2$ lateral heterostructure. b) AFM image of WS_2/$MoSe_2$. The red dotted line marks the area as WS_2. c) OM image of MoS_2/$MoSe_2$ lateral heterostructure. d) AFM image of MoS_2/$MoSe_2$. e) Low-magnification STEM image of $MoSe_2$ and WS_2 regions with a boundary. f) Zoomed-in image of the squared region in (e). g) Line intensity profiles of the line-marked region.

Table S1. Comparison of growth wafer size of $MoSe_2$ on various substrates reported in the literatures.

Reference	Method	Thickness	Growth wafer size	Substrate
[1]	CVD	monolayer	1×1 cm^2	SiO_2
[2]	selenization	few-layer	1×1 cm^2	SiO_2
[3]	selenization	few-layer	1×1.4 cm^2	SiO_2
[4]	NaCl-assisted CVD	monolayer	2.5×5 cm^2	SiO_2
[5]	CVD	monolayer	3×3 cm^2	sapphire
This work	CVD	monolayer	4 inch	sapphire

Reference

[1] X. Lu, M. I. B. Utama, J. Lin, X. Gong, J. Zhang, Y. Zhao, S. T. Pantelides, J. Wang, Z. Dong, Z. Liu, W. Zhou, Q. Xiong, *Nano Lett.* **2014**, *14*, 2419.

[2] T.-J. Dai, Y.-C. Liu, X.-D. Fan, X.-Z. Liu, D. Xie, Y.-R. Li, *Nanophotonics* **2018**, *7*, 1959.

[3] T.-J. Dai, X.-D. Fan, Y.-X. Ren, S. Hou, Y.-Y. Zhang, L.-X. Qian, Y.-R. Li, X.-Z. Liu, *J. Mater. Sci.* **2018**, *53*, 8436.

[4] D. Zhang, C. Wen, J. B. Mcclimon, P. Masih Das, Q. Zhang, G. A. Leone, S. V. Mandyam, M. Drndić, A. T. C. Johnson Jr., M.-Q. Zhao, *Adv. Electron. Mater.* **2021**, *7*, 2001219.

[5] M. Kim, J. Seo, J. Kim, J. S. Moon, J. Lee, J. H. Kim, J. Kang, H. Park, *ACS Nano* **2021**, *15*, 3038.
